# Outcomes of Patients with Heart Failure Hospitalized for COVID-19—A Study in a Tertiary Italian Center

**DOI:** 10.3390/diseases12120337

**Published:** 2024-12-21

**Authors:** Rossella Cianci, Mario Caldarelli, Pierluigi Rio, Giulia Pignataro, Marta Sacco Fernandez, Francesca Ocarino, Davide Antonio Della Polla, Francesco Franceschi, Antonio Gasbarrini, Giovanni Gambassi, Marcello Candelli

**Affiliations:** 1 Department of Translational Medicine and Surgery, Catholic University of Sacred Heart, 00168 Rome, Italy; mario.caldarelli01@icatt.it (M.C.); pierluigi.rio18@gmail.com (P.R.); francesca.ocarino01@icatt.it (F.O.); antonio.gasbarrini@unicatt.it (A.G.); giovanni.gambassi@unicatt.it (G.G.); 2 Fondazione Policlinico Universitario A. Gemelli, Istituto di Ricerca e Cura a Carattere Scientifico (IRCCS), 00168 Rome, Italy; giulia.pignataro@policlinicogemelli.it (G.P.); marta.sacco.fernandez01@icatt.it (M.S.F.); davideantonio.dellapolla@policlinicogemelli.it (D.A.D.P.); francesco.franceschi@unicatt.it (F.F.); marcello.candelli@policlinicogemelli.it (M.C.); 3 Department of Emergency, Anesthesiological and Reanimation Sciences, Catholic University of Sacred Heart, 00168 Rome, Italy

**Keywords:** COVID-19, heart failure, vaccination, sex differences

## Abstract

**Background:** Coronavirus Disease 2019 (COVID-19), triggered by SARS-CoV-2, has represented a global pandemic associated with an elevated rate of mortality, mainly among older individuals. The extensive pulmonary involvement by the viral infection might have precipitated pre-existing chronic conditions in this vulnerable population, including heart failure (HF). **Materials and Methods:** The aim of this retrospective, observational study was to assess the impact of COVID-19 in patients with a prior diagnosis of HF referred to the Emergency Department of the Agostino Gemelli University Hospital between March 2020 and January 2023. A total of 886 HF patients (444 men and 442 women, mean age of 80 ± 10 years) were identified. Patients were matched in a 1:1 ratio by gender, age, number of comorbidities (excluding HF), and vaccination status, using a propensity score matching (PSM) procedure. We compared the outcomes of 189 patients with a concomitant diagnosis of HF with those of 189 matched controls without HF. **Results:** Among patients with HF, there was a significantly higher prevalence of valvular disease (*p* = 0.004), atrial fibrillation (*p* = 0.003), use of anticoagulants (*p* = 0.001), chronic obstructive pulmonary diseases (*p* = 0.03), and chronic kidney disease (*p* = 0.001). In contrast, hypertension was more prevalent among controls than HF patients (*p* = 0.04). In addition, controls exhibited higher lymphocytes counts and a higher PaO_2_/FiO_2_ ratio compared to HF patients. During hospitalization, patients with HF were more frequently treated with high-flow nasal cannulas (*p* = 0.01), required more frequent admission to an intensive care unit (ICU) (*p* = 0.04), and showed a significantly higher mortality rate (*p* 0.0001) than controls. **Conclusions:** HF is an independent risk factor for ICU admission and death in COVID-19 patients.

## 1. Introduction

The global pandemic known as Coronavirus Disease 2019 (COVID-19), caused by the novel Coronavirus SARS-CoV-2, emerged in December 2019 and swiftly spread worldwide [1]. The clinical presentation of COVID-19 ranged from asymptomatic cases to severe conditions characterized by acute distress respiratory syndrome, mainly due to the extensive pulmonary involvement that was associated with elevated mortality rates. In Italy, a third of COVID-19 positive patients had at least one co-morbid condition, such as cardiovascular and respiratory chronic diseases, diabetes, metabolic disorders, neoplasms, obesity, renal disease, or other chronic illnesses [2]. 

Heart failure (HF) is a clinical condition caused by complex physiological processes that lead to a structural and functional failure of cardiac muscle [3]. The signs and symptoms of HF, particularly shortness of breath, fatigue, and fluid retention, reflect the most recent definition of the condition: “the inability of the heart to pump blood throughout the body as needed or to do so only by increasing filling pressures” [4].

Symptoms and exercise capacity are key to assessing the severity of HF and to monitoring the effectiveness of treatment. The New York Heart Association (NYHA) classification is commonly used, although the prognosis of HF is more accurately determined by both symptoms (NYHA class) and echocardiographic criteria [5].

Data from Europe and North America show a decline in the age-specific incidence of HF, with a significant shift toward HF with preserved ejection fraction, particularly in women, reflecting changes in the epidemiology of HF [6].

The incidence, prevalence, and mortality rates of HF are higher in blacks than in other racial or ethnic groups. The incidence and prevalence of HF are on the rise, primarily as a consequence of an aging population [6].

In this study, we analyzed the impact of COVID-19 in patients with HF referred to the Agostino Gemelli University Hospital—an established COVID-19 hospital—between March 2020 and January 2023. 

## 2. Materials and Methods

### 2.1. Study Design and Setting

We conducted a retrospective monocentric observational study on patients diagnosed with SARS-CoV-2 infection who were referred to the Emergency Room of a tertiary academic medical center (Fondazione Policlinico Agostino Gemelli—IRCCS in Rome). We retrieved all patients who were positive for the SARS-CoV-2 RT-PCR test on nasopharyngeal or oropharyngeal swabs and were hospitalized. We compared the clinical outcomes of COVID-19 in patients with a concomitant diagnosis of HF, who were matched to COVID-19 patients without a history of HF (control group) using a propensity score matching (PSM) procedure. In this analysis, controls were matched in a 1:1 ratio by gender, age, number of comorbidities, and vaccination status. Our main outcomes were in-hospital death, death 30 days from admission, and the need for admission to an intensive care unit (ICU). A multivariate logistic regression analysis was performed to assess the variable independently associated with the outcomes.

Additionally, we performed a secondary analysis within the HF patients to evaluate gender differences. We divided the HF patients by gender and assessed the presence of differences between the two groups using both univariate and multivariate analyses.

### 2.2. Inclusion and Exclusion Criteria

Inclusion criteria:

- Patients at least 18 years of age with a diagnosis of COVID-19 in the Emergency Room by an RT-PCR on a naso- or oropharyngeal swab;
- Patients who needed to be admitted to hospital;
- Patients with a documented diagnosis of HF;
- Patients with complete medical records, including laboratory tests, radiology imaging, and clinical.
- Exclusion criteria:
- Patients under 18 years old;
- Pregnant women.

### 2.3. Data Collection

For each patient, medical staff retrieved data via electronic health records on SARS-CoV-2 vaccination status, clinical characteristics, comorbidities, virus-related lung involvement (pulmonary embolism, need for oxygen supplementation), laboratory parameters, and pharmacological treatment. The valvular disease has been regarded as a comorbidity in patients who have undergone previous valve replacements—whether biological or mechanical—as well as those who have received transcatheter valve implantation and in cases where echocardiographic evaluations indicate moderate to severe stenosis or insufficiency. Participants were classified as unvaccinated if they had never received a COVID-19 vaccine, while those patients who received at least one dose of vaccine were considered vaccinated.

### 2.4. Ethical Issues

The study was approved by the local ethics committee of the Catholic University of Sacred Heart [29 April 2022—N 0014840/22]. The guidelines approved by the Italian Medicines Agency (AIFA) in December 2020 (https://www.aifa.gov.it/documents/20142/1269602/SOC_ospedaliera_09.12.2020.pdf/021a4ffe-7a80-32ed-ee9c-65a383ff1b47, accessed on 25 September 2024) and the updated version of October 2021 (https://www.omco.pd.it/modulistica-docman/comunicazioni-aifa/463-soc-ospedaliera-04-10-2021/file.html, accessed on 25 September 2024) were followed for in-hospital treatment. The study was conducted following the principles of the Declaration of Helsinki.

### 2.5. Statistical Analysis

Data are presented as mean ± standard deviation (when normally distributed) or median and interquartile range (when not normally distributed) for continuous variables and as numbers and percentages for categorical variables. For categorical variables, a comparison between groups was performed using the chi-square or Fisher’s exact test where appropriate. In the case of continuous variables, the comparison between groups was performed using the Student t-test for independent samples for normally distributed variables and with the Mann–Whitney U test in the other case. Initially, we performed two multiple logistic regressions using the need for ICU admission and death at 30 days as the independent variables to estimate the association with outcomes after adjusting for age, gender, and any variable with a *p*-value less than 0.15 in the univariate analysis. Subsequently, we matched, in a 1:1 ratio, patients and controls from our cohort of patients using a PSM. The PSM was calculated using a logistic regression multivariate analysis model. We used the nearest neighbor technique with no replacement. To avoid poor matching, we set the caliber size to 0.2. 

The results were reported using OR and 95%CI. Statistical analysis was performed using IBM SPSS version 20.0. We consider a *p*-value to be significant when it is lower than 0.05.

## 3. Results

### 3.1. Study Population Overall Results

A total of 886 patients (444 men and 442 women, mean age of 80 ± 10 years) were enrolled in our study. 295 patients presented with HF and 591 were controls without HF. HF patients showed a significantly higher number of comorbidities than controls, such as coronary heart disease (*p* = 0.007), valve disease (*p* = 0.0001), atrial fibrillation (*p* = 0.0001), chronic obstructive pulmonary diseases (COPD) (*p* = 0.006), and chronic kidney disease (*p* = 0.0001). Furthermore, HF patients used anticoagulants (*p* = 0.0001) more frequently at home and presented higher levels of BUN than controls (*p* = 0.0001). On the other hand, diseases such as inflammatory bowel diseases, autoimmune disorders, chronic neurological disorders (*p* = 0.0001), and hypertension (*p* = 0.007) were more common among controls. 

HF patients reported a significantly higher vaccination rate than controls: 44% of patients received at least one dose (*p* = 0.001), 18% two doses (*p* = 0.002), and 22% three doses (*p* = 0.0001). During hospitalization, HF patients received corticosteroids more frequently than controls (*p* = 0.0001). 

Table 1 summarizes the demographic, clinical, and laboratory data of the two groups.

### 3.2. Study Population Results After PSM

After PSM, the study sample included 378 patients (mean age 80 ± 10 years), of whom 203 (54%) were men. Over 90% of the patients had at least two concomitant chronic conditions, most commonly hypertension (69%), atrial fibrillation (32%), diabetes (32%), COPD (31%), coronary heart disease (30%), and active cancer (20%). One-third of the patients were obese and the mean BMI was 27 ± 5 kg/m^2^. Chronic pharmacological treatment included anticoagulants (36%) and ACEi/ARB (43%). 

As for the in-hospital treatment, anticoagulants were prescribed in 83% of patients, corticosteroids in 45%, remdesivir in 20%, monoclonal antibodies in 5%, and Tocilizumab in 4%. 

Among patients with HF, there was a significantly higher prevalence of valve disease (*p* = 0.004) atrial fibrillation (*p* = 0.003), and use of anticoagulants (*p* = 0.001), as well as COPD (*p* = 0.03) and chronic kidney disease (*p* = 0.001). In contrast, the prevalence of hypertension was higher among controls than in HF patients (*p* = 0.04). Moreover, controls had higher lymphocyte counts and a higher PaO_2_/FiO_2_ ratio than HF patients. 

Overall, during hospitalization, 58 patients (15%) were transferred to the ICU, and 75 (20%) died. Patients with HF were more frequently treated with high-flow nasal cannulas (18 vs. 9%; *p* = 0.01), more commonly required admission to ICU (19 vs. 12%; *p* = 0.04), and showed a higher mortality rate (39 vs. 1%; *p* = 0.0001) than controls.

In a multivariate logistic regression, ICU admission (Table 2) and death (Table 3) resulted strongly and independently associated with HF in COVID-19 patients. Similarly, HF was also independently associated with a composite outcome death and/or need for ICU admission (Table 4).

### 3.3. Sex Differences in COVID Patients with HF

A total of 295 COVID-19 patients with HF were analyzed for sex differences. Men were significantly younger than women (*p* = 0.0005) and had a higher prevalence of coronary heart disease (*p* = 0.04). In contrast, atrial fibrillation (*p* = 0.02) and Alzheimer’s disease (*p* = 0.01) were more common among women. Men used more angiotensin-converting enzyme inhibitors (ACEi) and angiotensin II receptor blockers (ARB) compared to women (*p* = 0.06) (Table 5). No differences were reported in SARS-CoV-2 vaccination rate between the groups. Compared to women, men showed a higher rate of admissions to ICU (*p* = 0.04), and oro-tracheal intubations (*p* = 0.02), and used high-flow nasal cannulas (*p* = 0.05) more frequently. In the multivariate analysis, men with HF showed significantly a higher age, lower LDH values, higher use of ACEi/ARB, and lower number of Alzheimer’s disease than women, as reported in Table 6. 

## 4. Discussion

The findings of the study reveal that among HF patients with COVID-19, the risk of death was nearly 40 times higher compared to controls. The clinical severity of the infection was higher, as testified by the higher number of admissions to the ICU and the significantly more frequent use of high-flow nasal cannulas. 

HF was the only independent variable for ICU admissions and deaths after adjustment for age, gender, number of comorbidities, and vaccination status.

Previous data indicate that among hospitalized patients with COVID-19, the prevalence of HF ranged from 4.9% to 13% [7] and that these patients are at an increased risk of mechanical ventilation and experience an extended stay in the ICU [8]. 

The detrimental impact of SARS-CoV-2 on the in-hospital prognosis of patients with HF can be attributed to different potential mechanisms. These include direct insult from the virus with an ensuing immune-mediated response, resulting in inflammation followed by myocardial injury, edema, and myocarditis. Additionally, demand ischemia may occur due to respiratory failure and hypoxemia. Post-viral autoimmune reactions can also be considered potential contributors [9].

The study by Guney et al. evaluated the neutrophil-to-lymphocyte-platelet ratio (N/LP) as an indicator for predicting ICU admission, the need for mechanical ventilation, and in-hospital mortality in COVID-19 patients [10]. In an analysis of 134 patients, higher N/LP values were associated with an increased risk of complications and death. The N/LP ratio proved to be a simple but promising marker for monitoring clinical risk in these patients [10].

Moreover, in another study, Guney et al. identified the C-reactive protein/albumin ratio (CAR) as a predictive indicator of in-hospital mortality in COVID-19 patients [11]. The study showed a strong association between higher CAR levels and increased risk of death.

In other studies, age, HF, and lack of vaccination were found to be independent factors for mortality. Several studies have documented the protective effect of vaccination against SARS-CoV-2 not only to prevent but also to reduce the severity of COVID-19 disease and improve the prognosis of hospitalized patients [12,13]. 

In our study, despite being younger, men with HF had a higher number of admissions to the ICU than women (*p* = 0.03). Increasing evidence suggests that being a man is a potential risk factor for both more severe clinical conditions and higher mortality rates [14]. In all countries reporting sex-disaggregated data, higher COVID-19 mortality rates have been consistently documented among men [14,15]. Women exhibit a higher count of CD4+T cells, a more potent CD8+T cell cytotoxic activity, and an increased B cell production of immunoglobulin than men [16]. As a result, women possess an enhanced ability to initiate humoral immune responses compared to men [17]. Women also generate high levels of type 1 interferon (IFN), in response to the detection of viral RNA by Toll-like receptor 7. This is crucial for an early and effective response in the context of COVID-19.

In our HF group, men used more ACEi/ARB agents than women. Prior studies have suggested that decreasing the expression of ACE2 may lower the vulnerability to SARS-CoV-2 infection in laboratory settings, within living organisms, and in human lungs and livers undergoing perfusion outside the body. This evidence has been corroborated by several model verification experiments [18]. RAAS inhibitors, ACEi and ARB, have conventionally served as primary medications for hypertension treatment [19]. Nevertheless, the utilization of ACEi/ARB in hypertensive patients with COVID-19 has generated debate and controversy. In a retrospective study, Zhang et al. illustrated that the continued administration of ACEi or ARB agents could potentially halt the progression from mild-moderate COVID-19 infection to more severe stages. The utilization of ACEi/ARB seems particularly advantageous for patients aged 60 years or older, with an even more pronounced benefit observed in those aged 80 years or older [20]. 

Obesity is an independent prognostic factor in COVID-19, and patients with a higher BMI are at increased risk for mechanical ventilation and mortality, compared to patients with normal BMI [21]. Obesity impairs breathing due to the restriction of lung expansion with reduced vital capacity, ventilation/perfusion mismatch, and subsequent hypoxemia. In addition, obesity is accompanied by chronic systemic inflammation and an increased expression of ACE2 that facilitates the SARS-CoV-2 entry into host cells [22]. Moreover, the COVID-19 lockdown has radically changed individuals’ habits by promoting sedentary behaviors, physical inactivity, and the consumption of unhealthy foods, as well as inducing stress and cortisol hypersecretion, thus representing a determinant of weight gain and obesity [23]. 

The study by Cinar et al. analyzed the prognostic nutritional index (PNI) as a predictor of in-hospital mortality in 294 COVID-19 patients at high cardiovascular risk [24]. Patients in the lowest PNI group (T1) exhibited a significantly higher mortality rate (11.2 times greater) than that of the highest PNI group (T3). The PNI was shown to be an independent and more effective indicator than albumin levels, lymphocyte count, and CURB-65 and 4C scores [24]. We hypothesized that multiple factors contribute to the increased risk of death in HF patients affected by COVID-19. On the one hand, the systemic and pulmonary effects of SARS-CoV-2 infection, including the release of inflammatory cytokines and the development of vascular micro-thromboses, may have a more severe impact on patients with reduced cardiac function due to heart failure compared to those with normal cardiac function [25].

Additionally, corticosteroids used in severe COVID-19 patients requiring oxygen supplementation—who are typically more critically ill and at a higher risk of death—may exacerbate heart failure and worsen prognosis. Glucocorticoids have been associated with both direct cardiotoxic effects and indirect adverse cardiovascular outcomes, including elevated blood pressure [26]. Animal studies further demonstrate that corticosteroid use can lead to systolic and diastolic dysfunction by disrupting calcium exchange processes and interfering with calcineurin signaling pathways [26,27].

## 5. Limitations

Our study has several limitations. As with any observational study, it cannot establish direct causal relationships, and the results may be influenced by unmeasured confounding factors. The focus on older patients hospitalized for COVID-19 limits the generalizability of our findings to younger individuals or those with different comorbidities. Despite using propensity score matching, residual selection bias may still exist, and the study may be affected by incomplete or inaccurate data, particularly regarding self-reported information, such as vaccination history. Therefore, further research is needed to confirm these findings and explore potential causal relationships.

## 6. Strengths

The use of propensity score matching and a sufficiently large sample size are the two main strengths of the study. These approaches helped mitigate the bias inherent in observational studies by reducing the influence of confounding variables.

## 7. Conclusions

In conclusion, in our cohort of hospitalized COVID-19 patients, HF appears to be an independent predictor for increased ICU admission and all-cause death. Vaccination reduces the severity of COVID-19 disease and improves the prognosis of hospitalized patients.

## Figures and Tables

**Table 1:** COVID-19 control group demographic, comorbidities, laboratory, and outcomes data compared to study group.

	Study Group			Propensity Matching Group		
	Controls	HF	*p*	Controls	HF	*p*
	(N = 591)	(N = 295)		(N = 189)	(N = 189)	
Demographic data						
Age (years, mean ± SD)	80 ± 10.1	80 ± 10.2	0.91	80 ± 10.6	80 ± 10.1	0.97
Men N (%)	296 (50.1)	148 (50.2)	0.95	103 (54.5)	100 (52.9)	0.76
Women N (%)	295 (49.9)	147 (49.8)	0.95	86 (45.5)	89 (47.1)	0.76
BMI (mean value ± SD)	26.8 ± 5.8	26.8 ± 6	0.89	27 ± 5.8	26.4 ± 5	0.23
Comorbidities N (%)						
Diabetes	203 (34.6)	94 (31.9)	0.42	63 (33.5)	59 (31.2)	0.63
Hypertension	430 (72.8)	189 (64.1)	0.007	140 (74.1)	122 (64.6)	0.04
Coronary heart disease	140 (23.7)	95 (32.2)	0.007	49 (25.9)	65 (34.4)	0.07
Cardiac valve disease	41 (6.9)	53 (18)	0.0001	16 (8.5)	35 (18.5)	0.004
Atrial fibrillation	137 (23.2)	122 (41.4)	0.0001	44 (23)	77 (40.7)	0.003
COPD	136 (23)	93 (31.5)	0.006	49 (25.9)	68 (36)	0.03
Active cancer	132 (22.3)	56 (19)	0.24	45 (23.3)	32 (16.9)	0.09
Pulmonary embolism	10 (1.7)	6 (2)	0.53	2 (1)	4 (2.1)	0.68
Chronic kidney disease	61 (10.3)	61 (20.7)	0.0001	15 (7.9)	37 (19.6)	0.001
Parkinson disease	42 (7.1)	12 (4.1)	0.07	10 (5.3)	8 (4.2)	0.6
Alzheimer’s disease	87 (14.8)	44 (14.9)	0.95	31 (16.4)	31 (16.4)	1
Obesity	180 (30.5)	80 (27.1)	0.12	68 (36)	56 (29.6)	0.19
Other chronic diseases *	559 (95.2)	47 (15.9)	0.0001	27 (14.2)	37 (19.6)	0.17
N. of comorbidities > 1	511 (86.6)	265 (89.8)	0.14	173 (91.5)	169 (89.4)	0.48
At-home treatment N (%)						
Anticoagulants	149 (25.2)	129 (43.7)	0.0001	52 (27.5)	83 (43.9)	0.001
ACEi/ARB	290 (49.5)	133 (45.1)	0.22	88 (46.6)	87 (46)	0.92
Laboratory Values (Mean value ± SD)						
BUN (mg/dL)	29.5 ± 24	36.4 ± 25	0.0001	26.9 ± 17	37.5 ± 26	0.001
LDH (UI/L)	334 ± 183	365 ± 298	0.11	318 ± 159	346 ± 194	0.13
CRP (mg/L)	72 ± 71	74 ± 68	0.7	70 ± 64	73 ± 70	0.59
Procalcitonin (ng/mL)	0.63 ± 3.2	1.93 ± 3.2	0.18	0.68 ± 3.4	0.8 ± 3.6	0.83
Lymphocytes (×10^9^/L)	1.1 ± 0.6	1.2 ± 1.7	0.64	1.19 ± 0.61	1.10 ± 0.49	0.03
D-dimer (ng/mL)	3106 ± 5995	3438 ± 5969	0.5	3207 ± 5364	3610 ± 6401	0.63
Fibrinogen (mg/dL)	503 ± 309	477±168	0.1	501 ± 170	473 ± 173	0.12
PaO_2_/FiO_2_	293 ± 153	279 ± 102	0.21	295 ± 89	269 ± 95	0.01
Vaccination N (%)						
At least one dose	124 (21.0)	130 (44.1)	0.0001	61 (32.2)	61 (32.2)	1
One dose	15 (2.5)	10 (3.4)	0.47	8 (4.2)	3 (1.6)	0.38
Two doses	60 (10.1)	52 (17.6)	0.002	27 (14.3)	26 (13.3)	0.96
Three doses	48 (8.1)	66 (22.3)	0.0001	26 (13.8)	31 (16.4)	0.84
Four doses	1 (0.2)	2 (0.7)	0.26	1 (0.5)	1 (0.5)	1
In-hospital treatment N (%)						
Corticosteroids	224 (38)	121 (41)	0.0001	88 (46.6)	85 (45)	0.76
Outcomes						
High-flow-nasal cannula N (%)	50 (8.4)	42 (14.2)	0.008	17 (9)	35 (18.5)	0.01
Non-invasive ventilation N (%)	39 (6.5)	20 (6.8)	0.9	16 (8.5)	18 (9.5)	0.72
Oro-tracheal intubation N (%)	21 (3.5)	16 (5.4)	0.18	6 (3.2)	11 (5.8)	0.21
ICU admission N (%)	77 (13)	47 (15.9)	0.24	22 (11.6)	36 (19)	0.04
Deaths N (%)	13 (2.1)	100 (33.9)	0.0001	2 (1.1)	73 (38.6)	0.0001

* Inflammatory bowel diseases, autoimmune disorders, chronic neurological disorders. COPD: Chronic obstructive pulmonary disease. BUN: Blood urea nitrogen, LDH: lactic dehydrogenase, CRP: C-reactive protein.

**Table 2:** Multivariate logistic regression for outcome ‘admission to ICU’ in patients with COVID-19.

Admission to ICU	*p*	OR (95% CI)
Age	0.000	1.058 (1.028–1.090)
Male sex	0.404	0.763 (0.404–1.441)
Vaccine	0.427	1.312 (0.671–2.564)
Comorbidities > 2	0.164	0.315 (0.062–1.602)
Heart failure	0.019	2.147 (1.136–4.058)
Obesity	0.144	1.599 (0.852–3.001)
Cardiac valvular disease	0.277	0.599 (0.238–1.508)
Atrial fibrillation	0.265	1.441 (0.758–2.741)
Chronic kidney disease	0.845	0.916 (0.382–2.198)
Hypertension	0.180	1.665 (0.790–3.509)

**Table 3:** Multivariate analysis for outcome ‘death’ in patients with COVID-19.

Death	*p*	OR (95% CI)
Age	0.037	0.964 (0.931–0.998)
Male sex	0.944	0.978 (0.522–1.831)
Vaccine	0.026	2.143 (1.094–4.199)
Comorbidities > 2	0.469	0.653 (0.207–2.065)
Heart failure	0.000	60.267 (14.269–254.543)
Obesity	0.881	0.948 (0.471–1.907)
Cardiac valvular disease	0.597	0.809 (0.368–1.777)
Atrial fibrillation	0.152	1.578 (0.845–2.948)
Chronic kidney disease	0.382	1.402 (0.658–2.990)
Hypertension	0.538	1.233 (0.633–2.399)

**Table 4:** Multivariate analysis for composite outcome ‘death and/or need for ICU admission’ in patients with COVID-19.

Death/Need for ICU	*p*	OR (95% CI)
Male Sex	0.56	0.86 (0.515–1.438)
Age	0.38	1.01 (0.986–1.038)
Vaccines	0.13	0.66 (0.381–1.130)
Comorbidity > 2	0.20	1.95 (0.702–5.440)
Heart failure	<0.0001	6.46 (3.710–11.238)
Obesity	0.18	1.45 (0.837–2.517)
Cardiac valvular disease	0.24	0.66 (0.323–1.335)
Atrial fibrillation	0.11	1.53 (0.904–2.599)
Chronic kidney disease	0.52	1.25 (0.631–2.481)
Hypertension	0.14	1.53 (0.868–2.692)

**Table 5:** Demographic, comorbidities, laboratory, and outcomes data of COVID-19 patients with HF, according to sex.

	Study Group	Men	Women	*p*
	(N = 295)	(N = 148)	(N = 147)	
Demographic data				
Age (years, mean value ± SD)	80.2 ± 10.2	78.12 ± 11.34	82.23 ± 8.53	0.0005
Vaccinated N (%)	130 (44.1)	58 (44.6)	72 (55.4)	<0.09
Unvaccinated N (%)	165 (55.9)	90 (54.5)	75 (45.5)	<0.09
Chronic heart failure	295 (100)	148 (100)	147 (100)	
BMI (mean value ± SD)	26.8 ± 6	26.1 ± 4	27.6 ± 7.4	0.08
Comorbidities N (%)				
Diabetes	94 (31.9)	46 (31.1)	48 (32.7)	0.77
Hypertension	189 (64.1)	100 (67.6)	89 (60.5)	0.2
Coronary heart disease	95 (32.2)	56 (37.8)	39 (26.5)	0.04
Cardiac valve disease	53 (18)	30 (20.3)	23 (15.6)	0.3
Atrial fibrillation	122 (41.4)	51 (34.5)	71 (48.3)	0.02
COPD	93 (31.5)	51 (34.5)	42 (28.6)	0.28
Active cancer	56 (19)	29 (19.6)	27 (18.4)	0.79
Other lung conditions *	32 (10.8)	17 (11.5)	15 (10.2)	0.72
Pulmonary embolism	6 (2)	2 (1.3)	4 (2.7)	0.41
Chronic kidney disease	61 (20.7)	33 (22.3)	28 (19)	0.74
Parkinson disease	12 (4.1)	6 (4)	6 (4.1)	0.99
Alzheimer’s disease	44 (14.9)	12 (8.1)	32 (21.8)	0.01
Obesity	80 (27.1)	34 (23)	46 (31.3)	0.11
Other chronic diseases **	47 (15.9)	25 (16.9)	22 (14.9)	0.65
N. of comorbidities > 1	265 (89.8)	136 (96)	129 (88)	0.24
At-home treatment N (%)				
Anticoagulants	129 (43.7)	58 (39.2)	71 (48.3)	0.11
ACEi/ARB	133 (45.1)	78 (52.7)	54 (36.7)	0.06
Laboratory Values (Mean value ± SD) NT-pro-BNP	15, 113 ± 9074	13,718 ± 4232	17,009 ± 12,579	0.25
BUN (mg/dL)	36.4 ± 25	40.1 ± 28	32.8 ± 21	0.01
LDH (UI/L)	365 ± 298	357 ± 354	373 ± 232	0.66
CRP (mg/L)	74 ± 68	75.63 ± 66	72 ± 69	0.7
Procalcitonin (ng/mL)	0.63 ± 3.21	0.73 ± 4	0.5 ± 2	0.62
Neutrophils (×10^9^/L)	7 ± 6	7.9 ± 7	6.1 ± 4.5	0.11
Eosinophils (×10^7^/L)	0.06 ± 0.16	0.04 ± 0.06	0.07 ± 0.22	0.22
Lymphocytes (×10^9^/L)	1.2 ± 1.7	1.3 ± 2.4	1.1 ± 0.6	0.4
D-dimer (ng/mL)	3438 ± 5969	3809 ± 6811	3075 ± 4955	0.4
Fibrinogen (mg/dL)	477±168	480 ± 176	473 ± 161	0.68
PaO_2_/FiO_2_	279 ± 102	278 ± 109	281 ± 96	1
In-hospital treatment N (%)				
Anticoagulants	251 (85.1)	123 (83.1)	128 (87.1)	0.28
Corticosteroids	121 (41)	64 (43.2)	57 (38.8)	0.43
Remdesivir	49 (16.6)	25 (16.9)	24 (16.3)	0.89
Monoclonal antibodies ***	16 (5.4)	10 (6.8)	6 (4.1)	0.31
Tocilizumab	8 (2.7)	6 (4)	2 (1.4)	0.16
Outcomes				
High-flow-nasal cannula N (%)	42 (14.2)	27 (18.2)	15 (10.2)	0.05
Non-invasive ventilation N (%)	20 (6.8)	11 (7.4)	9 (6.1)	0.65
ICU recovery N (%)	47 (15.9)	30 (20.3)	17 (11.6)	0.04
Oro-tracheal intubation N (%)	16 (5.4)	13 (8.8)	3 (2)	0.02
Deaths N (%)	100 (33.9)	51 (34.5)	49 (33.3)	0.83

* Pulmonary fibrosis, asthma, obstructive sleep apnea. ** Inflammatory bowel diseases, autoimmune disorders, chronic neurological disorders. *** casirivimab and imdevimab. COPD: Chronic obstructive pulmonary disease. BUN: Blood urea nitrogen, LDH: lactic dehydrogenase, CRP: C-reactive protein.

**Table 6:** Multivariate analysis in HF COVID-19 patients according to sex differences.

Sex	*p*	OR	95% CI	
			Min	Max
Age	0.014	1.049	1.010	1.089
LDH	0.055	1.002	1.000	1.004
BUN	0.024	0.984	0.970	0.998
ICU	0.563	1.374	0.468	4.032
At-home anticoagulants	0.488	0.769	0.366	1.615
ACEi/ARB	0.045	2.793	1.024	7.614
Atrial fibrillation	0.222	0.624	0.292	1.331
Other lung conditions *	0.094	2.897	0.833	10.069
Alzheimer	0.036	0.350	0.131	0.934
Oro-tracheal intubation	0.325	2.628	0.384	17.985
Monoclonal antibodies	0.194	3.226	0.551	18.905

* Pulmonary fibrosis, asthma, obstructive sleep apnea. Abbreviations: LDH: lactic dehydrogenase, BUN: Blood urea nitrogen, ICU: intensive care unit, ACEi: Angiotensin-converting enzyme inhibitors, ARB: Angiotensin II receptor blockers.

## Data Availability

Data are available from the corresponding author upon reasonable request.
